# Whole transcriptome data analysis of zebrafish mutants affecting muscle development

**DOI:** 10.1016/j.dib.2016.05.007

**Published:** 2016-05-12

**Authors:** Olivier Armant, Victor Gourain, Christelle Etard, Uwe Strähle

**Affiliations:** Institute of Toxicology and Genetics, Karlsruhe Institute of Technology, Campus Nord, PO box, Karlsruhe, Germany

**Keywords:** Genetics, mRNASeq, Zebrafish, Muscle mutant, Development

## Abstract

Formation of the contractile myofibril of the skeletal muscle is a complex process which when perturbed leads to muscular dystrophy. Herein, we provide a mRNAseq dataset on three different zebrafish mutants affecting muscle organization during embryogenesis. These comprise the myosin folding chaperone *unc45b* (*unc45b−/−), heat shock protein 90aa1.1* (*hsp90aa1.1−/−*) and the *acetylcholine esterase* (*ache−/−*) gene. The transcriptome analysis was performed in duplicate experiments at 72 h post-fertilization (hpf) for all three mutants, with two additional times of development (24 hpf and 48 hpf) for *unc45b−/*−*.* A total of 20 samples were analyzed by hierarchical clustering for differential gene expression. The data from this study support the observation made in Etard et al. (2015) [1] (http://dx.doi.org/10.1186/s13059-015-0825-8) that a failure to fold myosin activates a unique transcriptional program in the skeletal muscles that is different from that induced in stressed muscle cells.

**Specifications Table**

**Table:** 

Subject area	*Biology*
More specific subject area	*Developmental/Stem cell biology*
Type of data	*Transcriptome, figures, tables*
How data was acquired	*High-throughput RNA sequencing using Illumina HiSeq 1000*
Data format	*Processed*
Experimental factors	*Wild type siblings versus mutants*
Experimental features	*Comparison of mRNA from unc45b−/−, hsp90aa1.1−/−, ache−/− to their respective wild type siblings at 3 stages of development. Duplicates are used for each condition.*
Data source location	*Institute of Toxicology and Genetics, Karlsruhe Institute of Technology, Campus Nord, Eggenstein-Leopoldshafen, Germany*
Data accessibility	*Data are available with this article, and via NCBI׳s GEO accession number GEO: GSE74202* (*through the direct link*http://www.ncbi.nlm.nih.gov/geo/query/acc.cgi?acc=GSE74202)

**Value of the data**

• This dataset comprises the transcriptome analysis of the zebrafish mutants *unc45b−/−*, *hsp90aa1.1−/−* and *ache−/−* during embryonic development.
• It provides the list of regulated genes and associated Gene Ontology analysis of skeletal muscle cells under cellular stress and defective chaperoning activity.
• It is anticipated that this dataset can serve as a reference point for other analysis on myopathies.

## 1 Data

This data consists of 20 high-throughput sequencing samples of *unc45b−/−*at 24 hpf, 48 hpf and 72 hpf, as well as *ache−/−* and *hsp90aa1.1−/*− and their respective siblings at 72 hpf (*n*=2 for each genotype and stage) generated on an Illumina HiSeq 1000 [1]. The data are deposited under the Gene Expression Omnibus (GEO) number GEO: GSE74202 at http://www.ncbi.nlm.nih.gov/geo/query/acc.cgi?acc=GSE74202.

## 2 Experimental design, materials and methods

### 2.1 Production of mutants and experimental design

Homozygous mutant zebrafish embryos were produced from incrosses of identified heterozygote mutant carriers in the AB genetic background for the lines *unc45b+/−* [2], *hsp90aa1.1+/−* (referred here after as *hsp90a*) [3] and *ache*+/*−* [4]. Wild type and mutant siblings were identified at 72 hpf for *hsp90a−/−* and *ache−/−*, and at 24 hpf, 48 hpf and 72 hpf for *unc45b−/−* by their morphology under the binocular. Embryos were collected from several incrosses in two independent collections. About 20–50 manually dechorionated embryos of each genotype were collected in fish water and homogenized in 200 µL Trizol (Thermo Fisher) after removing fish water with a pipette. The extraction of total RNA was performed as described in the manufacturer׳s protocol, with the modification that an additional extraction with chloroform was performed before precipitation with isopropanol. Total RNA pellets were resuspended in 50 µl RNase-free water (Ambion). RNA integrity was checked by loading about 100 ng total RNA on a RNA6000 Nanochip using an Agilent 2100 Bioanalyser (Agilent Technologies). Samples showed no sign of degradation (RNA index number>9). The list of genotypes and stages of samples collected in the study are provided in Table 1.

### 2.2 Library preparation, quality control and data analysis

Sequencing libraries were prepared with the TruSeq RNA Library Prep kit v2 (Illumina), following manufacturer׳s protocol. Briefly, total RNA (1 µg) for each sample was used for poly(A) RNA selection using poly-dT coated magnetic beads followed by fragmentation. First strand cDNA synthesis was performed with the Superscript II (Thermo Fisher) using random hexamer primers. The cDNA fragments were subjected to end-repair and dA-tailing, and finally ligated to specific double stranded bar-coded adapters. Libraries were amplified by 12 cycles of PCR. The quality and concentration of the resulting sequencing libraries were determined on a DNA1000 chip using an Agilent 2100 Bioanalyser (Agilent Technologies). The mRNASeq libraries were sequenced at 7 pM on a HiSeq1000 device (Illumina) to generate 50 bp paired-end reads. Cluster detection and base calling were performed using RTA v.1.13 and quality of reads assessed with CASAVA v.1.8.1 (Illumina). The mapping was performed with TopHat version 1.4.1 [5], setting the distance between mates to 180 bp and a standard deviation of 80 bp. Other TopHat options were -butterfly-search -coverage-search -microexon-search -a 5 -p 5 -library-type fr-unstranded and using the known exon-exon junctions from Ensembl release 75. Quantification of the mapped reads was determined with HTSeq version 0.5.3p3 [6] using the options --stranded=no --mode=union and using the gtf file from Ensembl release 75.

**Fig. 1:**
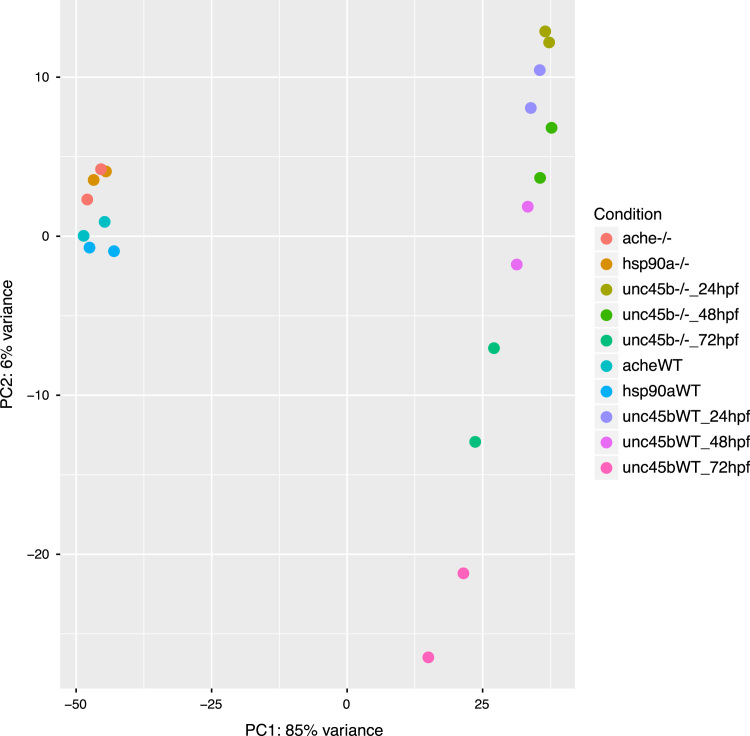
PCA plot. Visualization of the effects of experimental covariates and batch effect of the 20 samples analyzed by mRNASeq by their first and second principal components. Expression data were normalized using the regularized log transformation method from DESeq2.

**Fig. 2:**
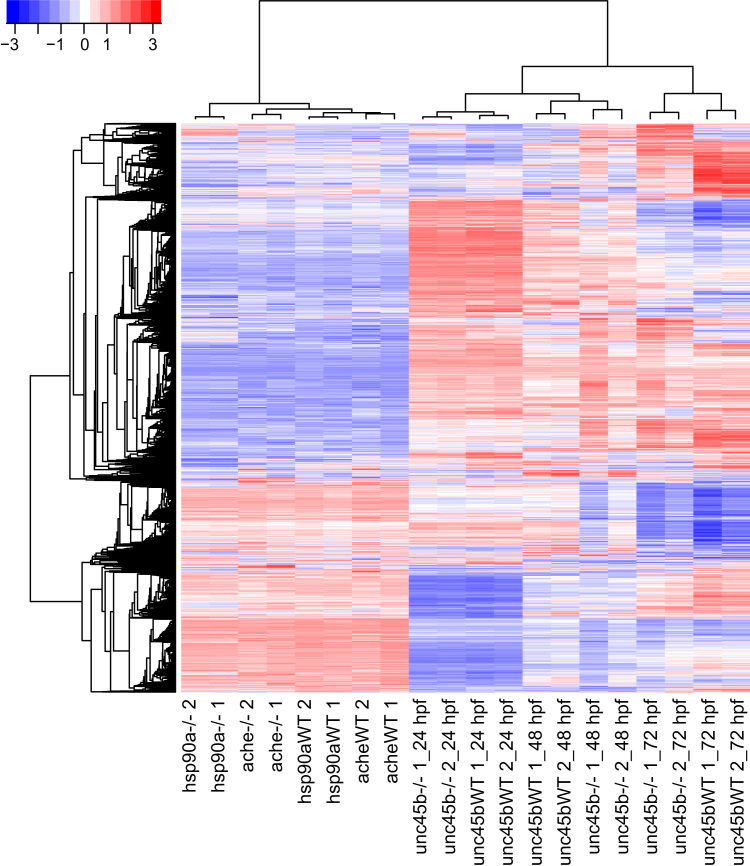
Hierarchical clustering of regularized log transformed data. 9453 genes with rlog>9 in at least one condition were selected and subjected to hierarchical clustering using Pearson׳s correlation and the complete-linkage method under Euclidean metric. Each row represents the expression level of a gene. High expression is symbolized by red color, white is moderate expression and blue low expression. The genotype of each sample is indicated. Mutant samples are indicated by (*−/−*) and wild type siblings by WT. Biological duplicates are indicated by the numbers 1 and 2.

**Fig. 3:**
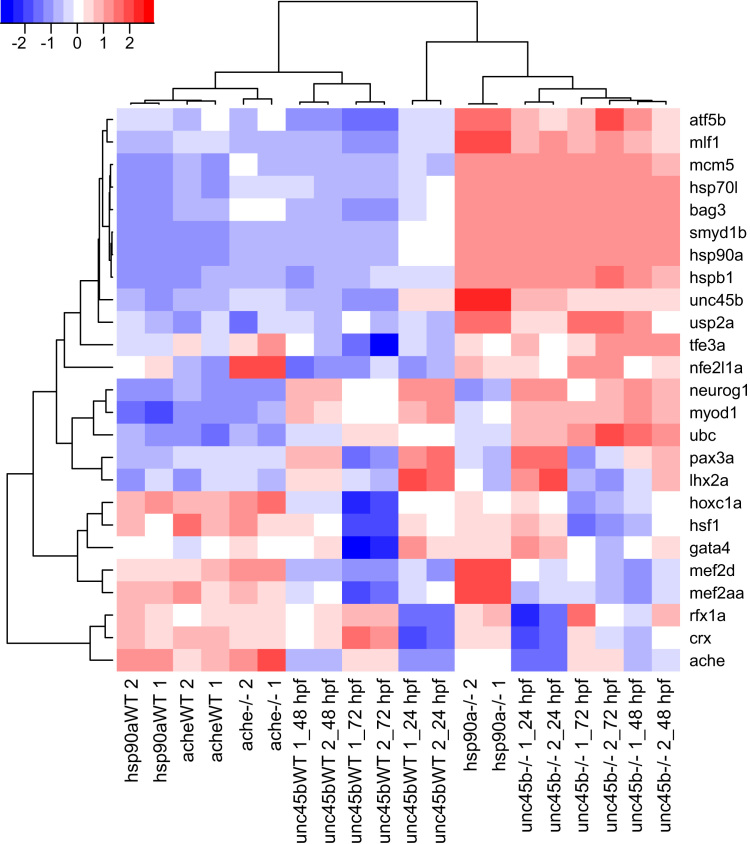
Heatmap of regularized log transformed data of 24 genes involved in muscle development and neurogenesis. Hierarchical clustering was performed with Pearson׳s correlation and the complete-linkage method using Euclidean metric on a selection of 24 genes involved in various biologic processes such myogenesis and neurogenesis (red: high expression, white: moderate expression, blue: low expression). The name of each gene is indicated. Mutant samples are indicated by (−*/−*) and wild type siblings by WT. Biological duplicates are indicated by the numbers 1 and 2.

The principal component analysis of the regularized log transformed (rlog) data from DESeq2 [7] shows that the biological duplicates are consistent and that the variance is mainly a factor of the stage and genotype (Fig. 1). The 9453 genes with rlog expression consistently>9 in at least one set of duplicate were subjected to hierarchical clustering with Pearson׳s correlation and the complete-linkage methods using the R packages *hclust* and *gplots* (Fig. 2). The hierarchical clustering of 24 selected genes involved in various biological processes such muscle development and neurogenesis shows specific patterns of expression depending on the genotype (Fig. 3).

**Fig. 4:**
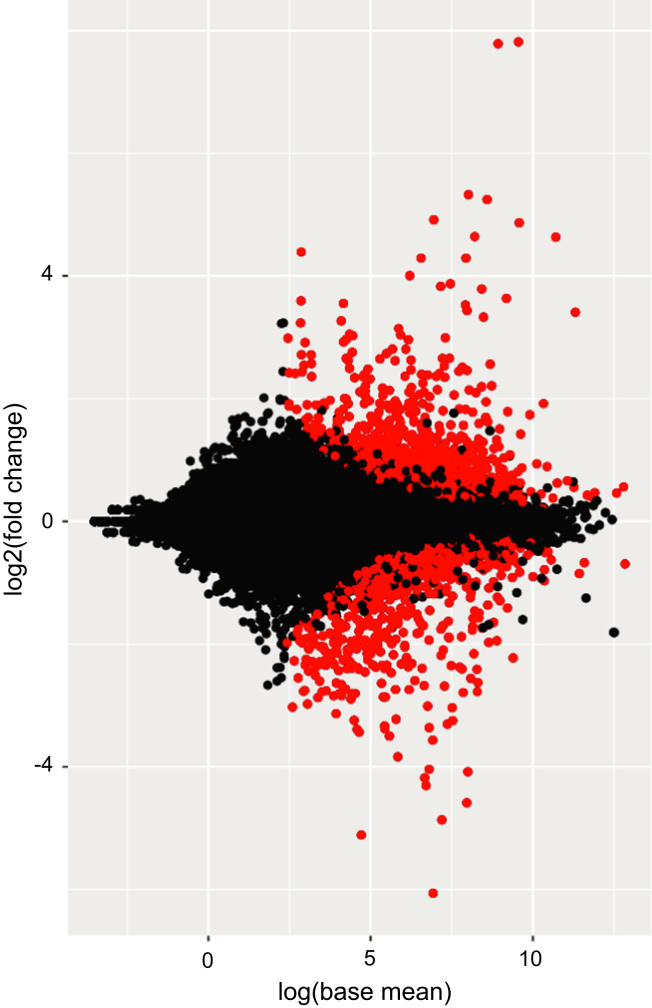
MA plot between the control and *unc45b−/−* conditions (72 hpf). For each gene the mean of the regularized log transformed expression is plotted against the log(fold change). Genes with a significant adjusted *p*-value (<0.01) are colored in red, and the others in black.

The differential gene expression of the binary comparisons between the mutant and the respective controls were made with the R package DESeq2. A MA plot for the analysis of the differential gene expression between control and *unc45b−/−* embryos at 72 hpf is shown (Fig. 4). For each gene, the mean expression was plotted against the logarithm of the fold change. Genes with significant deregulation are shown in red (adjusted *p*-value<0.01).

Zebrafish genes up-regulated or down-regulated in the comparative analysis between the *unc45b−/−* and control larvae at 72 hpf (adjusted *p*-value<0.01) were subjected to pathway enrichment analysis. First known human gene orthologues with at least 30% of identity or an orthology confidence score of 1 were obtained from Ensembl Compara [8] via Biomart (release 75) [9]. Then unique Ensembl human or zebrafish identifiers were used to obtain their associated Gene Ontology terms using the R package *biomaRt* [10]. Finally, enrichment of GO terms was assessed by computing *p*-values from a Fisher׳s exact-test (gene universe set to 18,000). A comparison between the top enriched GO terms with human and zebrafish genes up-regulated in the *unc45b*−/− compared to control at 72 hpf is given in Table 2, Table 3. The data show that pathways involved in muscle physiology and hypoxia are enriched in the set of genes up-regulated in *unc45b*−/− compared to the control at 72 hpf.

## Ethic approval

All experiments were made in accordance with the German animal protection standards and were approved by the Government of Baden-Württemberg, Regierungspräsidium Karlsruhe, Germany (Aktenzeichen 35-9185.81/G-137/10).

## Figures and Tables

**Table 1:** Description of the genotype and stage of development of the zebrafish embryos collected in the study. The mutants *ache−*/*−*, *unc45b−*/*−* and *hsp90a−*/*−* are caused by recessive mutations. The genotype of each sample is indicated. The mutant samples symbolized by (*−*/*−*) are homozygous mutant. The wild type siblings are annotated by WT and are constituted of embryos without any overt phenotype with a mixed genotype (+/+ or +/*−*). Biological duplicates are indicated by the number 1 and 2.

**Sample name**	**Genotype**	**Stage**	**Description**
ache−/− 1	*ache−/−*	72 hpf	Mutant *ache−/−* at 72 hpf, replicate 1
ache−/− 2	*ache−/−*	72 hpf	Mutant *ache−/−* at 72 hpf, replicate 2
hsp90a−/− 1	*hsp90a−/−*	72 hpf	Mutant *hsp90a−/−* at 72 hpf , replicate 1
hsp90a−/− 2	*hsp90a−/−*	72 hpf	Mutant *hsp90a−/−* at 72 hpf , replicate 2
unc45b−/− 1_24 hpf	*unc45b−/−*	24 hpf	Mutant *unc45b−/−* at 24 hpf, replicate 1
unc45b−/− 1_48 hpf	*unc45b−/−*	48 hpf	Mutant *unc45b−/−* at 48 hpf, replicate 1
unc45b−/− 1_72 hpf	*unc45b−/−*	72 hpf	Mutant *unc45b−/−* at 72 hpf, replicate 1
unc45b−/− 2_24 hpf	*unc45b−/−*	24 hpf	Mutant *unc45b−/−* at 24 hpf, replicate 2
unc45b−/− 2_48 hpf	*unc45b−/−*	48 hpf	Mutant *unc45b−/−*at 48 hpf, replicate 2
unc45b−/− 2_72 hpf	*unc45b−/−*	72 hpf	Mutant *unc45b−/−* at 72 hpf, replicate 2
ache WT 1	*ache+/−* ; *ache+/+*	72 hpf	Wild type siblings of *ache−/−* at 72 hpf, replicate 1
ache WT 2	*ache+/−* ; *ache+/+*	72 hpf	Wild type siblings of *ache−/−* at 72 hpf, replicate 2
hsp90a WT 1	*hsp90a+/+* ; *hsp90a+/−*	72 hpf	Wild type siblings of *hsp90a−/−* at 72 hpf, replicate 1
hsp90a WT 2	*hsp90a+/+* ; *hsp90a+/−*	72 hpf	Wild type siblings of *hsp90a−/−* at 72 hpf, replicate 2
unc45b WT 1_24 hpf	*unc45b+/−* ; *unc45b+/+*	24 hpf	Wild type siblings of *unc45b−/−* at 24 hpf, replicate 1
unc45b WT 1_48 hpf	*unc45b+/−* ; *unc45b+/+*	48 hpf	Wild type siblings of *unc45b−/−* at 48 hpf, replicate 1
unc45b WT 1_72 hpf	*unc45b+/−* ; *unc45b+/+*	72 hpf	Wild type siblings of *unc45b−/−* at 72 hpf, replicate 1
unc45b WT 2_24 hpf	*unc45b+/−* ; *unc45b+/+*	24 hpf	Wild type siblings of *unc45b−/−* at 24 hpf, replicate 2
unc45b WT 2_48 hpf	*unc45b+/−* ; *unc45b+/+*	48 hpf	Wild type siblings of *unc45b−/−* at 48 hpf, replicate 2
unc45b WT 2_72 hpf	*unc45b+/−* ; *unc45b+/*+	72 hpf	Wild type siblings of *unc45b−/−* at 72 hpf, replicate 2

**Table 2:** Human GO term enrichment. Human orthologues of zebrafish genes up-regulated in *unc45b*−/− at 72 hpf were used to query GO terms and assess pathway enrichment with a Fisher׳s exact-test.

**GO ID**	**GO Name**	**Fisher *p*-value**	**Genes**
GO:0001666	Response to hypoxia	1.47E−09	PPARA,RAF1,PLAT,SDC2,CITED2,MTHFR,CXCR4,PGF,ALKBH5,TGFB1,VEGFA,ABAT,PDLIM1,EPO,TGFBR3,ARNT,ALAS2,SMAD9,PDGFB, ANGPT2,LIMD1,MECP2,STAT5B,PLOD1
GO:0032355	Response to estradiol	7.56E−09	PTGS2,KCNJ11,TGFB1,RNF14,ASS1,TACR1,WNT7A,ANXA1,CASP9,NCOA1,PDGFB,IGFBP2, SLC25A36,GHR,STAT5B
GO:0030018	Z disc	2.42E−07	MYH6,XIRP2,PDLIM5,MYPN,SLC4A1,SYNPO2,JPH2,BIN1,SLC8A1,PGM5,MYL9,SYNPO2L,OBSL1
GO:0007596	Blood coagulation	4.44E−07	SLC7A8,PLEK,RAF1,PSAP,PLAT,SERPINE1,ITGA4,SOS1,PLA2G4A,LYN,FLNA,ANO6,TGFB1, PDE11A,GATA1,VEGFA,PDE10A,PDE2A,PIK3R5,L1CAM,ZFPM1, PRKG1,ITGB3,LRRC16A,MERTK,SLC8A1,PDGFB,ANGPT2,DOCK1,HBE1,GATA4,SERPINB2, PRKACB,SLC16A1,SLC3A2
GO:0030334	Regulation of cell migration	6.68E−07	ROBO4,NOTCH1,CXCR4,TGFB1,JAG1,LAMA3,UNC5C,LAMA2,HACE1,ITGB3,PTPN23,LAMA1
GO:0002040	Sprouting angiogenesis	7.57E−07	NOTCH1,ESM1,PGF,NRARP,VEGFA,JMJD6,GPR124
GO:0007507	Heart development	7.91E−07	TBX5,PPARA,PPARG,RAF1,CITED2,RBM20,ITGA4,SALL4,ACVR2B,NOTCH1,ZFP36L1, SOX9,JMJD6,TGFBR1,PAX3,WT1,GLI2,ZFPM1,PDGFB,GATA4,RBPJ,SOX18
GO:0009986	Cell surface	8.33E−07	PLAT,ITGA4,NOTCH1,CXCR4,GPRC5B,LRP4,ANO6,TIMP2,TGFB1,CSF1R,VEGFA,HBEGF,EPHA2,CLMP, TACR1,WNT7A,L1CAM,ANXA1,PTGFRN,TFRC,GPR124,EPO,ITGB3,TGFBR3,KCNH2,GREM1,SDC4, CNTNAP2,LRRC8A,MST1R,PDGFB,ANXA2,SPTB,GHR,FGFR3,SLC3A2
GO:0001837	Epithelial to mesenchymal transition	8.57E−07	SNAI1,NOTCH1,FLNA,TGFB1,SOX9,HMGA2,TGFBR1,TGFBR3,RBPJ
GO:0007220	Notch receptor processing	9.90E−07	NOTCH1,JAG1,NOTCH3,UBB,DLL4,DLL1

**Table 3:** Zebrafish GO term enrichment. Zebrafish genes up-regulated in *unc45b*−/− at 72 hpf were directly used to query GO terms and assess pathway enrichment with a Fisher׳s exact-test.

**GO ID**	**GO Name**	**Fisher *p*-value**	**Genes**
GO:0007219	Notch signaling pathway	1.62E−06	dll4,notch1a,notchl,jag1a,chac1,notch3,notch1b,dlc,dla,nrarpb,dld
GO:0002040	Sprouting angiogenesis	9.73E−06	dll4,cdh5,tinagl1,rbpjb,vegfaa,sdc2,nrarpb,jmjd6,cxcr4a
GO:0030218	Erythrocyte differentiation	2.17E−05	epb41b,tfr1a,slc4a1a,gata1a,alas2,gfi1b,stat5.1
GO:0035162	Embryonic hemopoiesis	5.73E−05	epb41b,tfr1a,slc4a1a,gata1a,alas2,sptb,irak3
GO:0030334	Regulation of cell migration	4.11E−04	robo4,LAMA3 (1 of 2),LAMA2 (2 of 2),lama1,itgb3a
GO:0048821	Erythrocyte development	1.24E−03	spi1b,gata1a,sptb,stat5.1
GO:0055002	Striated muscle cell development	1.60E−03	myf5,tcf3a,vangl2,cxcr4a
